# How Capital Endowment and Ecological Cognition Affect Environment-Friendly Technology Adoption: A Case of Apple Farmers of Shandong Province, China

**DOI:** 10.3390/ijerph18147571

**Published:** 2021-07-16

**Authors:** Hongyu Wang, Xiaolei Wang, Apurbo Sarkar, Fuhong Zhang

**Affiliations:** 1 Department of Economics & Management, College of Economics & Management, Northwest A&F University, Yangling 712100, China; wanghongyu@nwafu.edu.cn (H.W.); apurbo@nwafu.edu.cn (A.S.); 2 The Sixth Industry Research Institute, Northwest A&F University, Yangling 712100, China; 3 Department of Information Science and Engineering, College of Information Science and Engineering, Shandong Agricultural University, Tai’an 271018, China; 2018110568@sdau.edu.cn; 4 Department of Economics & Management, College of Economics & Management, Shandong Agricultural University, Tai’an 271018, China

**Keywords:** capital endowment, ecological cognition, environment-friendly technology, adoption level, Hackman model

## Abstract

Ever-increasing global environmental issues, land degradation, and groundwater contamination may significantly impact the agricultural sector of any country. The situation worsens while the global agricultural sectors are going through the unsustainable intensification of agricultural production powered by chemical fertilizers and pesticides. This trend leads the sector to exercise environmentally friendly technology (EFT). Capital endowment and ecological cognition may significantly impact fostering farmers’ adoption of environmentally friendly technology. The government also tends to change the existing policies to cope with ever-increasing challenges like pollution control, maintaining ecological balance, and supporting agricultural sectors substantially by employing ecological compensation policy. The study’s main objective is to explore the impacts of farmer’s ecological compensation, capital endowment, and ecological cognition for the adoption of EFT. The empirical setup of the study quantifies with survey data of 471 apple farmers from nine counties of Shandong province. The study used Heckman’s two-stage model to craft the findings. The results showed that 52.02% of fruit farmers adopted two environmentally friendly technologies, and 23.99% of fruit farmers adopted three forms of environmentally friendly technologies. At the same time, we have traced that the capital endowment, planting scale, family income, and technical specialization of fruit farmers significantly impact adopting EFT. The study also revealed that understanding ecological compensation policy has a significant positive effect on adopting environmentally friendly technology. Seemingly, ecological compensation policy has a specific regulatory effect on fruit farmers’ capital endowment and ecological cognition. Therefore, it is necessary to extend the demonstration facilities, training, and frequently arrange awareness-building campaigns regarding rural non-point source pollution hazards and improve the cognition level of farmers. The agriculture extension department should strengthen the agricultural value chain facilities to make farmers fully realize the importance of EFT. Government should promote and extend the supports for availing new and innovative EFT at a reasonable price. Moreover, cooperative, financial, and credit organizations need to lead for the smooth transition of EFT. The agricultural cooperatives and formal risk-taking networks should act responsibly for shaping the behavioral factors of farmers.

## 1. Introduction

China has gained relatively swift development in agricultural sectors by employing intense inputs within the last decade. It has a long history of given priorities for intensifying agriculture production to support the massive population with limited arable land [1]. For mitigating these challenges, widespread and overuse of chemical fertilizers and pesticides has been adopted by Chinese agriculture sectors [2]. However, China is achieving the challenges of food security by employing intensive agro-productivity powered by intensive interactions of chemical components, but those activities have drawn exacerbated controversies for maintaining the sustainable development goals set by the united nations (UN) [3]. The impermissible utilization of chemical components and overuse of natural resources resulted in extra production costs and staggering ecological issues from the soil, water, and air contaminations by greenhouse gas emissions [4,5,6]. These concerns are not only slowing down sustainable development goals achievement but also threatened human wellbeing and existence. Therefore, China is confronting significant sustainability issues as a densely populated and agriculture-based nation [7]. Intensive and chemical input-based agricultural production methods have been updated, and innovative eco-friendly technology is rigorously studied, introduced, and reviewed to reduce farm emissions from non-point sources and enhance its influence. As a result, Chinese farmers are trying to improve their environmental stand while sustaining or increasing crop production [8]. Global studies have shown that the usage of environmentally friendly technologies could be crucial to reduce the rural non-point source of pollution and improve the quality of products [6,9]. Seemingly, several eco-friendly technologies have been applied in horticultural production, such as soil testing and formula fertilization, organic fertilizer instead of chemical fertilizer, green prevention and control technology, soil improvement technology, and so on [10]. Moreover, it revealed a smooth progression from emphasizing scientific research facilities to participative field trials [11].

Interestingly, research has shown that by adopting environment-friendly technology, farmers can improve product quality, protect the ecological environment, and effectively integrate into the high-value industrial chain [12,13]. The continuous adjustment of the agricultural and industrial structure has forced more farmers to turn their limited land resources to planting high-yield and high-value products [14]. From the effect, the application of the technologies reduces the rural non-point source pollution. It also improves the quality of products conducive to obtaining the food quality and safety certification to lay a foundation for products to enter the high-end and dynamic consumption market [15,16]. The strategies of reducing the non-point cause of agricultural emissions are entirely satisfactory within demonstration zones and experimental stages, whether the effectiveness of those innovative tactics and the promotions and adoption intentions are not sufficiently explored yet [17]. Thus, the areas where small farms can practically use this advanced technology would have to be assessed appropriately. According to Grzelak et al. [18], resource endowments such as human and materials resources of capital approach to farming may impact fostering EFT. It is apparent that if a farmer has certain freedom and resource supports, it provides confidence for farmers to try many forms of potential technology [4]. Several pieces of research also indicate that farmers’ cognition level largely influences the farmers to adopt environmentally friendly technologies [19,20]. If farmers possess a positive attitude towards a certain EFT, it is easier for them to make decisions for adopting EFT in the future [21]. In terms of emerging countries, the ecological compensation policies may significantly impact the impacts of EFT [22]. Especially among the smallholder farmers, the impacts of such policies may have crucial impacts to build up a positive attitude and eventually improve their cognition level. Therefore, based on apple farmers’ actual situation in Shandong and from the capital endowment and ecological cognition prospects, this paper analyzes the impact mechanism of environment-friendly technology adoption. It further explores the mediating impacts of ecological compensation for shaping cognition and improving farmer’s capital endowment capabilities.

Related literature mainly focuses on influencing factors and adopting decision-making by using environment-friendly technologies [23]. Due to the difference in endowment, the heterogeneity of their technology adoption behavior is apparent, and the age factor is usually included in the estimation model first. For example, Wang et al. [20] provided a brief assessment on the adoption behavior of soil testing, and formula fertilization technology for grain crop farmers shows that the older the age, the lower the possibility of technology adoption, which is in line with the previous studies on Integrated Pest Management of vegetable farmers. However, Zhou et al. [24] also argued that experienced farmers might have a greater willingness to adopt water-saving and labor-saving technologies. Seemingly, Ma et al. [25] addressed that farmers with higher education tend to have a deeper understanding of using chemical products scientifically and rationally to reduce the impacts of excessive use and have a higher adoption rate of environment-friendly technologies.

Moreover, the promotions and guidance from suppliers also have a significant impact on practical usage of chemical products and technology adoption behavior of farmers [26], while the planting years, scale, number of laborers, and whether to join cooperatives also have an impact on the adoption behavior of environment-friendly technologies [27]. Interestingly, farmers’ new technology adoption decision-making is different from the adoption intention [19], which is a rational choice after comparing the expected cost and benefit [28]. Farmers show a low awareness of green prevention and control technologies such as biological pesticides, have uncertainty about the effect [25], and demand a profound paradox in the application, willingness, and behavioral changes [29]. Whether farmers adopt environment-friendly technologies depends on the potential performance that can be improved after technology adoption, such as market share, yield level, and cost–benefit comparison [30]. However, the government’s subsidies, for example, price concession for innovative machinery utilization, price and procurement subsidies on organic fertilizers (cash grant and interest-free loans), and pest control, facilitating low-interest loans, accelerated depreciation, and rent rebates (indirect subsidies) to farmers who adopt environment-friendly technologies could have a good effect on improving the adoption of environment-friendly technologies and controlling rural non-point source pollution [9].

The existing studies mainly focus on single technology adoption, centered within the crop farmers’ context. In contrast, a minimal number of publications have been traced that can quantify adoption behavior towards environmental-friendly technologies among apple or other orchard-based products [31]. Moreover, the research on the adoption behavior and degree of farmer’s environment-friendly technology around capital endowment, ecological cognition, and ecological compensation policy is relatively rare, quantifying the article’s strength and prime novelty. Therefore, for fulfilling the research mentioned above the gap, these articles used the survey of 471 apple farmers from nine counties (cities, districts) of Shandong Province, and adopted the Heckman sampling model to provide an in-depth assessment of the adoption behavior and measured the degree of adoption of environmentally friendly technologies by orchard farmers from the perspective of capital endowment and ecological cognition.

The article is designed as follows: Section 1 comprised the introduction and theoretical baseline. Section 2 described a brief overview of the data sources and theoretical outline. Section 3 outlined the variables and research approaches, whereas Section 4 denotes the results and analytical framework. Section 5 comprised the discussion and Section 6 explored the conclusions of the study and policy recommendations.

## 2. Methodology

### 2.1. Data Sources

**Figure 1:**
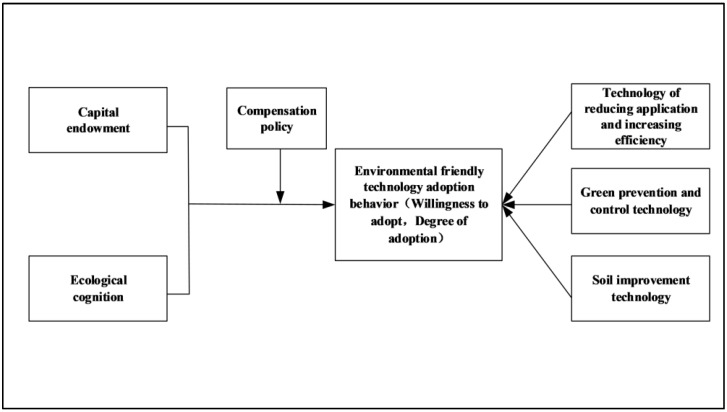
Theoretical model for the adoption of environment-friendly technology by farmers. Here, capital endowment and ecological cognition have been set as independent variables and “willingness to adopt” and “adoption” as dependent variables. In addition, compensation policy is composed of moderating variables.

The empirical data were collected through the field survey among the apple orchard farmers listed in the “National Research Center for Apple Engineering and Technology (NRCAET), Shandong Agricultural University,” situated in the most specialized apple production counties of Shandong province from December 2018 to January 2019. The surveyed regions were covered by nine major apple production counties (Penglai, Laiyang, Qixia, Haiyang, Longkou, Zhaoyuan, Zibo, and Linyi). Whereas we randomly selected 5-6 apple-growing townships from each county, each township selects a core apple-growing village. Finally, we randomly selected 10–15 apple growers from each selected village with sufficient communication skills to answer the questionnaire. A total of 500 questionnaires were distributed, and 471 valid questionnaires were collected, with an effective rate of 94.20%. Shandong is one of China’s largest apple-growing areas, and it is one of the largest exporter provinces in China, having strong market competitiveness within South Asia and Europe. However, due to the excessive application of fertilizers and pesticides, the local agricultural and environmental pollution problems are very prominent, so it is pertinent to study environmentally friendly technologies by apple growers. The nine counties surveyed in this article are also the core clusters of apple growers in Shandong. Thus, the data of the growers in these nine counties are representative in terms of the high demand of chemical usage, production rate, market values, and trends of synthetic pesticide usage of the selected area. Before the survey, a pilot test was conducted within the targeted areas to grasp the basic characteristics of the targeted area and respondents. Moreover, before the formal interviews were taken, the investigator briefly explained the content of the questionnaire, which might have influenced the high response rate. Figure 1 represents the theoretical framework adopted by the study.

### 2.2. Demographic Profile of the Respondent

The respondents are primarily middle-aged farmers, whereas 57.96% are over 50 years old, and 79.9% had junior high school education. Moreover, the planting scale is smaller than the average planting scale in these scales; 74.31% of them are less than 1.4 acres. On the other hand, the degree of specialization is high, 87.69% of apple production income accounts for more than 60% of the total income, and 80.3% joined the agricultural cooperatives. On the whole, the sample farmers are representative. The results show that the adoption rate of soil improvement technology is relatively high, reaching 87.26%. Among those, Penglai City, Qixia City, Yiyuan County, and Mengyin County are covered around 90% because they are situated within the pilot areas to replace chemical fertilizer with organic fertilizer. Simultaneously, reducing application and efficiency increasing technology has been found moderate, about 54.26%. It could have happed due to the favorable subsidy policy introduced by China. The results showed that the adoption level of reducing application and efficiency increasing technology in the above four pilot counties is higher than average.

In contrast, the adoption level of green prevention and control technology (biological control, physical trapping) is relatively low, only 18.47%, which may have happened for relatively high cost and slow effect of this technology, and the low recognition of the effect of this technology. Besides, 52.02% of the fruit farmers adopted two environmentally friendly technologies (soil improvement technology, reduction, and efficiency technology, green prevention, and control technology), 23.99% of the fruit farmers adopted three environmentally friendly technologies (soil improvement technology, reduced application efficiency technology, green prevention, and control technology). Therefore, it can be seen that fruit farmers’ subjective willingness to adopt environmentally friendly technology is more optimistic. However, the willingness and acceptance rate of green prevention and control technology in environmentally friendly technology is relatively low.

### 2.3. Theoretical Basis and Research Hypothesis

#### 2.3.1. The Direct Impact of Capital Endowment and Ecological Cognition on Farmer’s Adoption of Environment-Friendly Technology

The environment-friendly technologies in this paper mainly refer to the technologies of reducing and increasing efficiency of chemical fertilizer (soil testing and formula fertilization, water, and fertilizer integration technology), green prevention and control technology (artificial release of natural enemies, physical trapping), soil improvement technology (using organic fertilizer to replace chemical fertilizer, garden grass). Farmers need to invest in improving the human and material resources in adopting environment-friendly technology. EFT can be termed as applying knowledge that leads the sectors to enjoy not harming or less harming farming practices in agriculture. In the context of the study, we chose the three types of technologies as EFT, (i) technologies regarding reducing application and increasing efficiencies, (ii) green preventions and control technology, and (iii) soil improvement technology. Low-input technologies could act as a crucial EFT as the technology allows farmers to practice lower resource usage by increasing firm utility management while not compromising productivity [32]. Seemingly, green prevention and control technology directly allows farmers not to use or reduce the chemical intersection by employing bio-based organic components or integrated pest management practices to control the pests and diseases of the farm [33]. Finally, eco-friendly soil-management practices positively address serious environmental issues and maintain productivity [34,35].

When farmers make decisions, they are usually constrained by their capital endowment. The capital endowment has a significant impact on the farmer’s behavior choice and decision-making [36]. Due to the limitation of capital endowment, farmers may show lower adoption behavior when making adoption decisions [37]. On the contrary, if farmers possessed a sound capital endowment, it would be beneficial to them to take more risks and implement their innovativeness [38]. Thus, the study put forward Hypothesis 1 as (H1) capital endowment positively impacts the farmer’s adoption of environment-friendly technology. Seemingly, according to the planned behavior theory, farmers’ decisions will also be affected by subjective cognition [39]. In production, farmers will get an idea about the surrounding ecological environment, which will encourage farmers to make different decisions to adapt to the ecological environment changes [40,41]. Moreover, farmers’ cognition of changes positively impacts their living environment and eventually shaped the adoption behavior of environment-friendly technologies [42,43]. Additionally, farmers’ awareness of environmental risk positively impacts their adoption of environment-friendly behavior [44]. Therefore, capital endowment and ecological cognition will affect farmers’ adoption of environment-friendly technologies. Based on the above analysis, the author puts forward Hypothesis 2 as (H2) ecological cognition has a positive impact on the farmer’s adoption of environment-friendly technologies.

#### 2.3.2. Impact of Ecological Compensation Policy on Farmer’s Adoption of Environment-Friendly Technology

As a rational decision-maker, whether farmers finally decide to adopt new technologies is usually rational after comparing the expected costs and benefits. Therefore, if the government can provide certain policy subsidies to farmers (for example, price concession for innovative machinery utilization, subsidies on organic fertilizers, and pest control), it will help them eliminate their endowments’ restrictions and urge farmers to adopt environment-friendly technologies [38,39,40] actively. Additionally, ecological compensation (see above and references [22,45]) policy improves farmers’ awareness of environmental protection policies, thus promoting their enthusiasm to adopt environment-friendly technologies because farmers will adjust their production behavior according to agricultural subsidy policies [46]. Thus, the study put forward Hypothesis 3 as (H3) ecological compensation policy has a regulatory effect on farmers’ capital endowment and ecological cognition.

## 3. Variables and Research Approaches

### 3.1. Variables

#### 3.1.1. Dependent Variable

The dependent variables are “willingness to adopt” and “adoption” of environment-friendly technologies. The measurement of “willingness to adopt” uses the binary valuation method. To measure the “adoption” of environment-friendly technology, the values are 1–3 according to the number of types adopted by farmers to reduce application efficiency technology, green prevention and control technology, and soil improvement technology. If the farmers adopt any two or more of the above three technologies, the willingness to adopt environment-friendly technologies is relatively positive, and the value is 1. On the contrary, it is not active enough to adopt environment-friendly technology if the value is 0. Thus, the farmers’ willingness to adopt environment-friendly technologies indicates their views for the ecological management of orchards, and the degree of adoption of environment-friendly technologies indicates the efficiency of technology adoption.

#### 3.1.2. Independent Variables

This paper selects age, education, labor force, and duration of farming as the human aspects of capital endowment, which comprises exploring the local and international scholars within similar approaches (for more details, please see references [47,48,49]). In addition, the study selects planting scale, quality of agricultural pieces of machinery, income, and specialization as a material aspect of the capital endowment. Meanwhile, the respondents’ cognition on the harm of excessive fertilization, the cognition of soil environmental protection policy, and the cognition of environment-friendly technology to improve the ecological environment was selected to represent the ecological cognition.

#### 3.1.3. Moderating Variables

This paper selects the impact of ecological compensation policy as the moderating variable. The attitude and willingness of farmers to adopt environment-friendly technology will affect the ecological compensation policy and its implementation after technology adoption. We can learn from the relevant research results of Zhang Yu et al. [50] and Huang Xiaohui et al. [51]. The influence of compensation policy is represented by the degree of understanding, the satisfaction with ecological compensation policy, and the benefit to ecological compensation policy. The definition and descriptive statistics of variables are shown in Table 1.

#### 3.1.4. Research Approaches

Theoretically, in terms of adopting environment-friendly technology, in most cases, farmers will face a dilemmatic situation of whether they are willing to adopt it or not [52,53,54]. Previous studies mainly involved the issue of willingness to adapt [55,56,57], but not enough attention was paid to the degree of the farmer’s adoption intentions. From the perspective of subjective adoption intention, the first issue is whether the farmers are willing to adopt environment-friendly technologies, and if the farmers’ subjective willingness is not favorable, they will not fully adopt the three environment-friendly technologies. While the Heckman two-stage model can control the selectivity deviation caused by unobservable factors [58,59], it can deal with effect evaluation based on binary selection [60,61]. Therefore, this paper uses Heckman sampling to solve effect evaluation based on binary selection, as suggested by Lambrecht et al. [62].

Moreover, Heckman’s two-stage model can effectively solve the two-stage characteristics [63,64] of environmentally friendly technology adoption and is conducive to unbiased estimation [65]. The specific steps of the study are as follows: in the first stage, a probit selection model is established. The probit selection model is used to estimate the possibility of selection bias and calculate the inverse mills ratio (IMR). The function of IMR is to calculate a value for each sample to correct the sample selection bias. If the IMR is greater than 0, it indicates a selective bias in the sample. In the second stage, the estimated IMR of the first stage is put into the regression model of the second stage together with other variables by using the selective sample observations. Thus, the selection model’s self-selection problem was modified in the first stage and reflected by IMR in the second stage. After that, with the help of the Heckman sample selection model, the article estimates the farmers’ subjective willingness to adopt environmentally friendly technology $y_{1i}$ and the degree of farmers’ adoption of environmentally friendly technology $y_{2i}$ and verifies the hypothesis. On this basis, the study constitutes the interaction among the degree of ecological compensation, capital endowment, and ecological cognition to verify whether they significantly impact EFT adoption. Finally, the degree of farmers’ understanding, satisfaction, and benefits of ecological compensation policy were calculated and averaged. Then, the average value was used as the grouping standard, and those below and above the average value were divided into a group to examine the regulatory role of ecological compensation policy and test its robustness.

Although Heckman’s two-step selection model can effectively solve the endogenous problem caused by sample selection, it is very consistent with the two-stage technology adoption process [66]. However, there may still be data quality problems in data collection due to farmers’ lack of cooperation, and the model itself cannot solve these problems [67]. In addition, another major limitation of this methodology is that it is unable to carry out extensive statistical inference, the research sample is based on a specific region, and the research object is relatively single [68]. Finally, Heckman’s two-step selection model can only solve the endogenous problem caused by sample selection but cannot solve missing variables and reverse causality caused by other possible factors [69].

The sample selection model is used to deal with the above problems, which is as follows:

(1) $$y_{1i}=X_{1i}\alpha +\mu_{1i};y_{1i}=\{\begin{array}{ll} 1 & y_{1i}^{*}>0\\ 0 & y_{1i}^{*}\leq 0 \end{array}$$

(2) $$y_{2i}=X_{2i}\beta +\mu_{2i};y_{2i}=\{\begin{array}{ll} b & y_{1i}>0\\ 0 & y_{1i}\leq 0 \end{array}$$

(3) $$\begin{array}{l} E\left(y_{2i}|y_{2i}=c\right)=E\left(y_{2i}|y_{1i}^{*}>0\right)=E\left(X_{2i}\beta +\mu_{2i}|X_{1j}\alpha +\mu_{1i}>0\right)\\ =E\left(X_{2i}\beta +\mu_{2i}|\mu_{1i}>-X_{1i}\alpha \right)=X_{2i}\beta +E\left(\mu_{2i}|\mu_{1i}>-X_{1i}\alpha \right)\\ =X_{2i}\beta +\rho \sigma \mu_{2}\lambda \left(-X_{1i}\alpha \right) \end{array}$$

Equation (1) represents the selection equation, and Equation (2) represents the resulting equation. Where *i* represents the number of the grower; $y_{1i}$ represents the willingness to adopt environmentally friendly technologies; $y_{2i}$ represents the degree of adoption of technologies, which are the dependent variable; $X_{1i}$ and $X_{2i}$ are the independent variables of the two equations; $y_{1i}^{*}$ is latent variables that cannot be observed; *b* indicates that several technologies were adopted. The selection mechanism is as follows: only when $y_{1i}^{*}$ > 0, $y_{2i}$ can be observed. Meanwhile, α and β are the parameters to be estimated $\mu_{1i}$ and $\mu_{2i}$ are residual, consistent with the normal distribution.

The conditional expectation of farmers’ adoption of environment-friendly technology is as follows:

In (3), where λ is the inverse mills ratio function. While ρ is the coefficient of correlation of $y_{1i}$ and $y_{2i}$, when ρ = 0, it means that $y_{2i}$ it will not be affected by $y_{1i}$, and when ≠ 0, $y_{2i}$ will be affected by $y_{1i}$. Therefore, there is an ample selection bias, σ denotes the standard deviation.

## 4. Results and Analysis

### 4.1. Impact Analysis of Farmer’s Willingness to Adopt Environment-Friendly Technologies

The estimated results in Table 2 show that the ownership of agricultural machinery, the cognition of environment-friendly technology to improve the ecological environment, and the understanding of farmers’ ecological compensation policy have passed the positive significance test of 10%. It shows that the more agricultural machinery owned by farmers in the capital endowment, the higher the intensity of adopting environment-friendly technology. In ecological cognition, the higher the awareness of environment-friendly technology to improve the ecological conditions, the easier it is to adopt environment-friendly technology, which further leads farmers to improve the ecological environment of the orchard. As for the impact of ecological compensation policy, farmers are more likely to adopt environment-friendly technology to improve the ecological environment. Farmers’ understanding of ecological compensation policies positively affects their willingness to adopt environment-friendly technologies. Farmers need to pay a specific cost to adopt environmentally friendly technologies. If they cannot get the compensation, farmers’ views to adopt environment-friendly technologies will be affected. Therefore, understanding and mastering the ecological compensation policies can promote them to adopt environment-friendly technologies.

### 4.2. Analysis of the Impact of the Adoption of Environment-Friendly Technologies

From the adoption degree perspective, the planting scale, family income level, and a specialization degree in capital endowment have significant positive effects on farmers’ adoption of environment-friendly technologies. The larger the planting scale is for adopters, the higher the utilization efficiency of adopting environment-friendly technologies will be, and the adoption cost will be relatively reduced. In contrast, the high family income level has been found crucial for farmers to adopt environment-friendly technologies. If the farmer possessed higher income and specialized farming, the more likely they could be willing to adopt the environment-friendly technology [21,70,71]. Thus, Hypothesis 1 holds, but Hypothesis 2 has not been quantified. Besides, the understanding degree of ecological compensation policy significantly positively affects the adoption degree of farmers’ environment-friendly technology [22,72]. It indicates that an acceptable ecological compensation policy can enhance farmers’ willingness to adopt environment-friendly technology and help to improve the degree of farmers’ adoption of environment-friendly technology. From the perspective of the interaction between ecological compensation policy and farmers’ capital endowment and ecological cognition, the interaction coefficient of farmers understanding of ecological compensation and planting scale, family income level and specialization degree, soil environmental protection policy cognition, and environmental improvement effect cognition all have a positive impact on the adoption of environment-friendly technology at the level of 10%. The results show that the ecological compensation policy has a specific moderating effect on farmers’ capital endowment and ecological cognition.

## 5. Discussion

To further verify the robustness of the estimation results, this paper measures the ecological compensation policy variables and calculates the average value of the impact of ecological compensation policy on farmers by calculating the degree of understanding, satisfaction, and benefits of ecological compensation policy, which are parallel with Zhang Yu et al. [50] and Huang Xiaohui et al. [51]. Then, the average values are used as the grouping standard, and the groups below the average value and higher than the average value are divided into a group. The Heckman model was then used to quantify the influence of capital endowment and ecological cognition on the willingness and degree of adoption of environment-friendly technology in the two groups. Finally, the ecological compensation policy’s regulatory effect was investigated by examining the significant changes of different variable coefficients in the two groups [45,46,73]. The specific analysis results are shown in Table 3.

From the perspective of capital endowment, the group estimation results show that the influence coefficient of planting scale, family income level, and specialization degree on farmer’s adoption of environment-friendly technology has passed the significance test of 5%, and the regression results are consistent with the estimation results as shown in Table 2. Further, the coefficients of the ecological compensation policy are affected by the more extensive the scale of planting area, the higher the level of family income and the degree of specialization of farmers, the higher the enthusiasm of farmers to adopt environment-friendly technology, and the more comfortable to benefit from the ecological compensation policy, which is supported by Zhang et al. [74], Liu et al. [75] and Ke-Guo [76].

Seemingly, from the perspective of ecological cognition, the improved cognition effect of environment-friendly technology has a significant impact on farmers’ adoption of environment-friendly technology at the level of 10%, and the coefficient level of the high group is affected by the ecological compensation policy greater than that of the low group. It further verifies that the ecological compensation policy has a certain regulatory effect on farmers’ ecological cognition. Thus, the findings are verified by the study of Cai and Zhang [77], Home et al. [78], and Xuehai et al. [79]. However, it is worth noting that farmers’ ecological cognition has no significant impact on adopting environment-friendly technologies. This shows that the ecological compensation policy is conducive to help the majority of farmers to improve their awareness regarding improving the ecological environment [80,81,82]. Only if most farmers can acquire benefit from the process of technology adoption will they be attracted more towards the positive impact on the improvement of environment-friendly technology adoption in the future [78,83]. The assumption is also supported by Yuanquan and Wangsheng [84] and Dezdar [85]. Therefore, the government should continue to strengthen the ecological compensation policy and provide full support to enhance the awareness-building activities such as training facilities, boost the demonstration process, and massive circulation of the advantages of new and improved eco-friendly technologies.

## 6. Conclusions

The environmentally friendly technology adoption may crucial for fostering sustainable development goals set by the United Nations (UN). The adoption decision is a complex and dynamic phenomenon that could be affected by several factors. As per the core economic thought, farmers could adopt the EFT if they possessed enough capital endowment and the possibilities of economic benefit. Moreover, the proactive policy supports may also have significant impacts to improve the adoption tendencies. Within these circumstances, the article has been quantified by three pillars—capital endowment, ecological cognition, and ecological compensation policy and evaluated the impacts of those to foster environmentally friendly technology under the premise of maximizing profit. More specifically, the article explored the impacts of capital endowment and ecological cognition for facilitating EFT employing green preventions and control, efficient soil improvement, and low input production technologies. It also evaluated the underlying factors of human and material aspects of capital endowments for EFT adoption. Further, it also covered the factors associated with adoption willingness and degree of adoption to understand better the willingness and the actual adoption intensity of EFT.

The article utilized a dataset of 471 apple farmers extracted from a survey from nine counties of Shandong province to craft its findings. Overall, farmers positively responded to adopting environment-friendly technologies. However, there were significant differences in adopting the three kinds of environment-friendly technologies. The soil improvement technology was triggered the highest by comprising the adoption level of 87.26%, the middle rate of application was found for reduction and efficiency enhancement technology (54.26%). However, the lowest was found at 18.47% in terms of green pest control and management technologies. Whereas 52.02% of farmers adopted two kinds of environment-friendly technologies, 23.99% of farmers adopted three environment-friendly technologies. The study traces a positive connection between capital endowment and ecological cognition and reveals significant moderating effects of ecological compensation policy for facilitating EFT. From the perspective of capital endowment, the planting scale, family income level, and specialization degree have significant positive effects on adopting environment-friendly technology. The understanding of ecological compensation policy has a significant positive impact on farmers’ adoption of environment-friendly technology. Hence, the article’s findings will help formulate and implement relevant agricultural policies as it provided a theoretical basis for adopting environment-friendly technologies for orchards, especially apple farmers. Furthermore, this article is helpful to further research to understand the mechanism of the adoption of environmentally friendly technologies.

This paper draws the following policy recommendations: (i) as human capital is an integrated part of the capital endowment, the human capital empowering tactics such as training and demonstration facilities should be extended. Furthermore, to improve the farmer’s materials aspects of capital endowment, modern machinery and improved technologies should be introduced to harness the potentialities of modern science and technology. (ii) It is necessary to strengthen the information circulating facilities and education regarding rural non-point source pollution and the hazards of those within rural communities, to improve the cognitive level of the majority of farmers to participate in green development. Awareness-building campaigns should also be implemented to make farmers fully realize the importance of adopting environment-friendly technology in improving the ecological environment of the orchard, improving land fertility, and improving fruit quality. In addition, the potentialities of EFT should be highlighted among rural farmers, which will eventually improve the cognition level of farmers. (iii) Ecological compensation should be utilized by extending the existing agricultural subsidy policy for green development. Especially, instead of providing chemical fertilizer subsidies, the government should implement rewards programs for those who intend to or already utilize organic-based fertilizers and pesticides. (iv) The financial institutions and farmer’s organizations such as agricultural cooperatives should provide more financial support introducing new and innovative technologies, which could play a significant role in shaping cognition and improving access towards material aspects of capital endowments. Productivity may be increased by using environmentally friendly technology, which is also viable towards the farm’s profitability. Not essentially all the profitable technologies must be adopted by the farmers since barriers to practice innovative technologies and market uncertainty for environmental attributes interlinked with green technologies limit their effectiveness. The adoption and diffusion of alternative practices are also influenced by the factors such as the size of the farm, economic risk, and geographical location. It should be one of the crucial issues for policy consideration.

Apart from the monetary tools (direct subsidies), the government should emphasize indirect subsidies (insurance, low-interest loans, accelerated depreciation, rent rebates). The direct and indirect benefits of environmentally friendly technology, such as enhancing the ecological environment and biodiversity of the orchard, improving soil fertility and fruit quality, should also be highlighted and demonstrated among the farmers. Moreover, a sound interaction of e-commerce and value chain network facilities should also be implemented to make farmers fully utilize the betterment of modern science and technology. The social supports and obligations should also be prioritized, aligned with the other monetary incentives. The formal and informal risk-sharing organizations should also prioritize their scheme regarding risk minimizations and diffusions. This might be helpful to improve the cognition level and willingness of farmers to adopt environment-friendly technology.

Although the study revealed that the ecological compensation policy is helpful to trigger the majority of fruit growers to improve their understanding regarding EFT, how and to what extent the compensation policy triggers the adoption of EFT should be explored more distinctively. For example, if potential researchers could have traced whether there are any specific effects of several subsidies (subsidies per hectors or subsidies regarding modern equipment) for adopting EFT. Furthermore, the study focuses only on a single region with specific types of farmers; if more depth studies with several regions are combined, it would be more interesting. In addition, the social network will have a certain impact on the adoption of environment-friendly technology by influencing farmers’ information acquisition; the potential researchers should include this crucial factor into the core variable. Finally, as there are differences in the suitable orchard planting environment and planting scale of various environment-friendly technologies, further studies should trigger the specific planting environment and planting scale of various environment-friendly technologies combined with the social network in the future.

## Figures and Tables

**Table 1:** Variable definition and descriptive statistics.

Variables	Meaning and Assignment	AVG	Standard Deviation	Minimum	Maximum
Dependent variable					
Y1: The willingness	Adopting (two or more) positive = 1; otherwise = 0	0.51	0.49	0	1
Y2: Adaptation	Adoption	2.06	0.78	0	3
Capital endowment					
X1: Sex	F = 0; M = 1	0.37	0.48	0	1
X2: Age	By 2017	51.03	9.48	25	85
X3: Education	Years of education	7.64	5.22	0	11
X4: Duration of farming	Duration of farming	27.63	5.07	4	58
X5: Scale	AVG of the area in 2017	4.13	0.48	1	12
X6: Labor	Labors in family	3	1.49	0	12
X7: Machinery	Machinery	0.48	0.65	0	4
X8: Family income	income in 2017	10.52	0.95	7.60	13.30
X9: Specialization	Proportion to total income in 2017	68.47	7.89	60	86
Ecological cognition					
X10: Awareness of the hazards of over chemicals use	Do not know = 1; have heard of = 2; know something = 3; know very well = 4	1.83	1.02	1	3
X11: Awareness of soil environmental protection policy	Do not know = 1; have heard of = 2; know something = 3; know very well = 4	2.53	0.70	1	3
X12: Awareness on effect of environment-friendly technology	No = 1; little effect = 2; large action = 3; great effect = 4	2.28	0.50	1	4
The Impact of Environmental Policy					
X13: Understanding of ecological policy	1 = totally do not understand, 2 = do not understand, 3 = general, 4 = understand, 5 = fully understand	3.38	0.87	2	5
X14: Satisfaction with ecological policy	1 = very dissatisfied, 2 = not very satisfied, 3 = general, 4 = satisfied, 5 = very satisfied	3.18	2.04	1	5
X15: Benefit	1 = significant decrease, 2 = slight decrease, 3 = constant, 4 = slight increase, 5 = obvious increase	3.42	0.98	1	5

**Table 2:** Model regression results.

Variable	Willingness to Adopt		Degree of Adoption	
	Coefficient	Standard Error	Coefficient	Standard Error
x1 Sex	−0.214	0.150	−0.082	0.075
x2 Age	−0.006	0.008	−0.002	0.004
x3 Education	0.036	0.021	0.003	0.009
x4 Duration of farming	0.005	0.012	0.034	0.005
x5 Scale	−0.012	0.046	0.002 *	0.020
x6 Labor	−0.076	0.047	0.011	0.026
x7 Machinery	0.138 *	0.080	0.019	0.040
x8 Family income	0.011	0.078	0.059 *	0.035
x9 Specialization	−0.005	0.010	0.011 ***	0.004
x10: Awareness of excessive use	−0.011	0.072	−0.036	0.031
x11: Awareness of soil protection policy	0.140	0.103	0.215	0.052
x12: Awareness on improving effect	0.113 *	0.146	0.019	0.069
x13: Awareness of ecological compensation	0.111 *	0.082	0.053 *	0.040
x14: Satisfaction with ecological compensation	−0.072	0.080	−0.057	0.038
x15: Satisfaction with ecological compensation	0.083	0.076	0.015	0.037
x13: Awareness of ecological compensation * x5 Scale	—	—	0.029 *	0.020
x13: Awareness of ecological compensation * x8 Family income	—	—	0.042 *	0.155
x13: Awareness of ecological compensation * x9 Specialization	—	—	0.011 *	0.017
x13: Awareness of ecological compensation * Awareness of soil protection policy x13: Awareness of ecological compensation * x12: Awareness on improving effect	— —	— —	0.277 * 0.302 *	0.151 0.166
The constant	1.295	1.306	1.694 ***	0.575
Log-likelihood	−440.6671			
Wald chi2(15)		22.41		

Note: *, **, and *** represent significant at 10%, 5% and 1% confidence levels, respectively.

**Table 3:** Regression results of the robustness test.

Variable	High Group on Ecological Compensation		Low Group on Ecological Compensation	
	Willingness to Adopt	Degree	Willingness to Adopt	Degree
	Coefficient	Coefficient	Coefficient	Coefficient
X5: Scale	0.008 *	0.035 **	−0.002	0.032 *
	(0.016)	(0.208)	(0.074)	(0.020)
X7: Machinery	0.137 **	0.012	0.116	0.052
	(0.210)	(0.056)	(0.144)	(0.056)
lnX8: Family income	0.028	0.042 **	−0.160	−0.013
	(0.100)	(0.247)	(0.133)	(0.055)
X9: Specialization	0.009	0.008 **	−0.016	0.004 **
	(0.013)	(0.206)	(0.017)	(0.007)
X12: Cognition of improving environmental effect	0.078 **	0.087 *	−0.030	0. 017
	(0.019)	(0.012)	(0.252)	(0. 016)
The constant	−0.361	2.124 **	6.272 **	0.236
	(1.767)	(0.876)	(2.518)	(1.082)
Log likelihood	−266.0604		−177.2996	
Wald chi2(15)		24.46		27.46

Note: *, **, and *** represent significant at 10%, 5% and 1% confidence levels.

## Data Availability

The associated dataset of the study is available upon request to the corresponding author.
